# Measuring Health Literacy in Basic Education in Finland: The Development of a Curriculum- and Performance-Based Measurement Instrument

**DOI:** 10.3390/ijerph192215170

**Published:** 2022-11-17

**Authors:** Anna-Mari Summanen, Juhani Rautopuro, Lasse Kannas, Leena Paakkari

**Affiliations:** 1 Faculty of Sport and Health Sciences, University of Jyväskylä, 40014 Jyväskylän yliopisto, Finland; 2 Finnish Institute for Educational Research, University of Jyväskylä, 40014 Jyväskylän yliopisto, Finland

**Keywords:** curriculum, health literacy, school-age children, measurement, basic education, health education

## Abstract

This paper describes the development of an objective curriculum- and performance-based health literacy (HL) measurement instrument to assess Finnish 9th graders’ learning outcomes in the school subject termed Health Education (HE). There were four phases: (i) construction of the theoretical framework for the measurement, (ii) item generation, (iii) the field test (*n* = 252), and (iv) item analysis and item selection for the main study, in which 3652 ninth grade pupils (aged 15–16) participated. Initially, 303 HL test items were formulated, of which 107 were tested in two different field test versions. Both versions exhibited high reliability as measured by Cronbach’s alpha coefficient. The main study contained 55 items. Testing and item analysis enabled the development of a comprehensive competence- and curriculum-based HL measurement instrument for school-aged children. Measurement of HL in schools provides information for national policies, and for developing HE as a school subject.

## 1. Introduction

The foundations of health literacy (HL), health behavior, and overall health and wellbeing are built during childhood and youth [1,2,3]. Schools constitute an important arena for promoting HL [4,5,6] because they reach the majority of school-aged children over a long period of time [7]. Hence, the school is potentially the single most significant institution for increasing HL among adolescents [8]. According to the WHO [8], health literacy competencies should be developed “first and foremost through the school curriculum”. If HL is included in national school curricula it has the potential to decrease health disparities caused by differences in HL within countries [7]. This is especially the case if the national curriculum is combined with the missions and approaches of other stakeholders, including the government, NGOs, health authorities, and the media [9].

Given the positive associations between high HL and favorable health outcomes [10,11,12], the monitoring of HL is particularly important from a public health perspective. Moreover, when HL is learned through a specific curriculum with explicit HL learning objectives, it can be seen as an important academic competence and educational outcome *per se*. HE is a stand-alone subject in basic education and upper secondary education in Finland. This is a very different situation from many other countries, where HE is integrated within other school subjects. One can argue that the existence of HE as a statutory and compulsory subject highlights the relevance of HE.

In this context, the monitoring of HL among pupils is particularly valuable, since (among other benefits) it produces knowledge on how well they have reached the learning objectives defined in the school curriculum. This was the case in Finland in 2013, when a national assessment was conducted on the learning outcomes of the school subject labeled HE. The pupils in the assessment were 9th graders, aged 15–16 years.

This paper describes the development of a curriculum- and performance-based assessment of the learning outcomes of Health Education (considered as a distinct school subject) in basic education, organized by the Finnish National Agency for Education (EDUFI). During the last decade, many new instruments have been developed to measure HL among children and adolescents, and the measurement of HL has increased in the school context [12,13,14]. However, the majority of these instruments have focused on subjective HL [15,16] rather than objective HL. Furthermore, curriculum-based instruments are lacking.

## 2. The National Assessment of Learning Outcomes in the Finnish Context

From 1998 onwards, EDUFI has monitored how well pupils in basic education have succeeded in reaching the learning objectives laid down for various subjects in the national core curriculum. Summative assessments take place in each school subject at least once during the national core curriculum period, i.e., about once in ten years, at the end of basic education. The assessment of HL as a learning outcome of HE was undertaken when the subject had been a stand-alone subject in schools for almost a decade, i.e., in the spring of 2013. This was the first and only time that EDUFI assessed HE learning outcomes in basic education. The assessment focused on the learning of 9th graders (pupils aged 15–16) at the end of their final semester in basic education. The assessment was also the first examination of curriculum- and performance-based HL among school-aged children in Finland based on a nationally representative sample.

The assessment of learning outcomes is based on long-established national protocols in Finland. These nationally representative and sample-based assessments, conducted at the end of basic education, are designed to map and measure pupils’ knowledge, skills, and attitudes. The purpose of the assessment (involving an independent and systematic evaluation by an external institution) is to provide information for national and local policy- and decision-making with regard to schools. The results of learning outcomes are used to monitor equality in education in terms of gender, the official language of instruction of the schools (Finnish or Swedish), and the various regions. They can also assist in developing education and improving conditions for learning [17].

The assessment of learning outcomes in Finland includes characteristics that distinguish it from other countries. There are no national annual final examinations at the end of basic education in Finland. Nor are there rankings of schools, pupils, or teachers based on success in examinations. Although there are mandatory assessments of learning outcomes in a range of subjects in the national core curriculum [18], including in HE, the assessment system is based on the principle of development (in education) rather than control.

In Finland, HE has been a statutory, stand-alone, and obligatory school subject in basic education and upper secondary education for almost 20 years. The overall aim of HE as a school subject is to develop health-literate pupils [19]. Starting from 2004, every pupil in basic education in grades 7–9 (aged about 12–16) has had to have three courses (each of 38 h) in HE. By contrast, in general upper secondary education there is one obligatory course (38 h) in HE, in addition to a minimum of two optional courses. The subject of HE is also taught for younger learners (grades 1–6, aged about 7–12) as part of the subject called *environmental studies*, which includes also biology, geography, physics, and chemistry.

The position of HE as a separate and statutory school subject in Finland is fairly unique. In many other European countries, HE is integrated within other school subjects, projects, or classes [20,21,22]. The Finnish national HE curriculum describes the general aims for the subject, the objectives for the instruction, plus the learning criteria for a grade of 8 (grade scale 4–10). Note also that HE teachers are required to have a university-level education.

The aim of this paper is to describe the development of a comprehensive, theory-based, and curriculum-based instrument for measuring objective HL among children in the school context, as part of the assessment of learning outcomes in HE.

**Figure 1:**
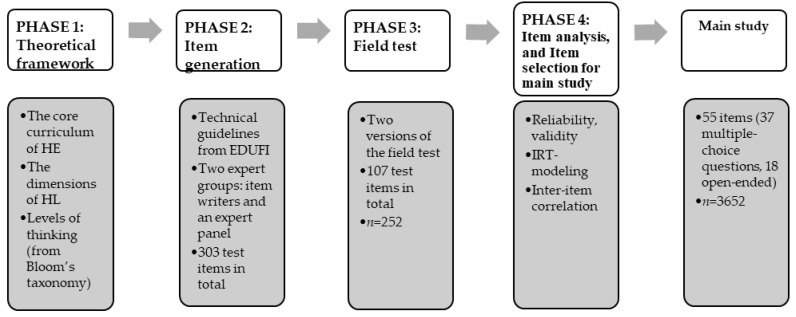
The phases in developing an HL measurement instrument for school-aged children (aged 15–16).

The development of the curriculum- and performance-based measurement instrument comprised four main phases: (i) creating a theoretical framework for the measurement, (ii) item generation, (iii) setting up a field test, and (iv) item analysis and item selection for the main study. The procedure followed the general principles for measurement development laid down by EDUFI [23]. The phases in developing the measurement instrument are summarized in Figure 1.

### 2.1. Creation of the Theoretical Framework for the Measurement (1st Phase) 

The theoretical framework for the HL item generation followed several themes: the national core curriculum for HE (including goals, core content areas, and criteria defined for HL final assessment numerical grade 8), the Finnish National Agency for Education [24], the dimensions of HL [25], and the levels of thinking, drawn from Bloom’s taxonomy [26].

The assessment was primarily based on the goals of the HE curriculum (see Appendix A), and especially on the final assessment criteria for achieving numerical grade 8 (scale 4–10), corresponding to level “good”. Such goals include, for example, that pupils should know how to care for themselves and their health, or that they should be able to give examples of how to promote health and safety in their own local environment [24]. Furthermore, the test was based on four core HE content areas: (i) *growth and development* (e.g., the life span of the individual, physical, psychological, and social growth and development, and taking care of one’s health); (ii) *health in choices in daily living* (e.g., nutrition health, sexual health, smoking, alcohol and drug abuse, the most common infectious diseases, traffic safety, solving conflicts and talking about worrisome issues); (iii) *resources and coping skills* (e.g., health, functional abilities as a resource, emotions, and interaction skills); (iv) *health, society, and culture* (e.g., national diseases, environment and health, and the rights of children and young people).

The conceptualization of HL as operationalized by Paakkari and Paakkari [25] was also used as a theoretical framework. According to them, a pupil’s competence regarding health and wellbeing (i.e., health literacy) is composed of five core components, namely (i) theoretical knowledge as an ability to remember basic health-related topics and conceptual models, (ii) practical knowledge (i.e., skills) as an ability to put theoretical knowledge into practice, (iii) critical thinking as an ability to critically evaluate the certainty of knowledge and to argue for one’s own perspectives, (iv) self-awareness as an ability to reflect on one’s ways of thinking, behaving, and feeling, plus metacognitive skills, and (v) citizenship as an ability to reflect on the consequences of one’s own ways of thinking and behaving on other people and on nature, and to participate in promoting the collective good.

The levels of thinking from Bloom’s taxonomy were used to cover a broad range and various levels of thinking, namely remembering, understanding, applying, analyzing, evaluating, and creating [26]. In the assessment, the latter three of these (analyzing, evaluating, and creating) were combined into one, to summarize general higher-order thinking.

### 2.2. Item Generation (2nd Phase)

Item writers (two HE school teachers and two academics from the university HE teacher training program) contributed to development of the items. The development process was guided by an expert panel (one methodologist from EDUFI and three university-level specialists in HE). The expert panel formulated the guidelines for the project based on the core curriculum and the established practices for EDUFI assessments. The item writers were asked to generate and categorize items within the HE core curriculum [24], and to consider also the dimensions of HL [25] plus levels of thinking from Bloom’s taxonomy [26].

In addition, EDUFI technical guidelines for preparing achievement tests [23] were considered. Based on these guidelines, about half of the total scores should come from closed-ended items. In practice, this means that about two thirds of the items should be closed-ended (as in multiple-choice questions) and one third of the items should be open-ended (requiring students to answer at greater length). Through the use of open-ended items, it is possible to assess pupils’ own thinking on the contents of HE, and their higher-order thinking (particularly via asking for explanations regarding responses). Moreover, according to the technical requirements, there should be an equal number of items per each of the four core HE content areas.

The items had to represent various levels of difficulty, covering easy, medium, and difficult content. Moreover, according to the guidelines [23], the level of difficulty (the solution percentage) of the main study as a whole should be between 60 and 65 percent. The first assessment of the difficulty level was conducted by the item writers and the expert panel, and the final assessment, applying statistical tests, was based on the solution percentages of the items in the field test phase. Interim knowledge of the difficulty level of the items available for selection for the main study was obtained from the field test. In total, 303 test items were generated. Out of these, 107 were chosen for the field test, based on the criteria outlined above. These were then divided into two test versions (i.e., two instruments). Using two test versions guaranteed the testing of a broad selection of items before selecting the final test items. The versions included four linking items (i.e., the same items in both test versions), which allowed for comparison of the levels of difficulty between the field test versions. Their purpose was also to check the feasibility of the items in both field test versions.

### 2.3. Field Test (3rd Phase)

Both test versions were tested in 10 upper secondary education schools (five general upper secondary schools, and five schools of vocational education and training) in different parts of Finland. The field test was carried out on students (about 16 years old) in the first year of secondary education school, as these students had recently completed the HE syllabus in basic education and thus resembled closely (in age and education) the students to be tested in the national assessment. The actual target group (9th grade pupils) could not be selected for the field test, since they had not yet completed three quarters of their 9th grade studies at the time of the field test. The field test sample included a total of 252 students (general upper secondary schools, *n* = 119; schools of vocational education and training, *n* = 133).

Two test versions were prepared for the field test. The item writers and expert panel selected the items for the tests. At this point, items found to be unclear, complex, too simple, too difficult, too similar in content to another item, or irrelevant, were removed. In the end, both field test versions included 56 items. These covered various core content areas of HE, five dimensions of HL, the four levels of thinking, a range of difficulty levels, and different item types (closed-ended and open-ended items). A two-stage stratified sampling method was used to select the schools from different parts of Finland. The students completed the test anonymously during one or two lessons. Half of the students in each teaching group responded to field test version A, and half to version B. The main tasks for the field test were to find the best-performing assessment items, to check for ambiguities and appropriateness in the wording, and to check the time used to complete the test.

### 2.4. Item Analysis and Item Selection for the Main Study (4th Phase)

In analyzing the items, several statistical methods were used to judge the quality of the test items [27]. In this study, all the student responses were checked and scored by two content experts from EDUFI. At the same time, the coverage and comprehensibility of the guidelines were reviewed. The data for the field test versions were analyzed using SPSS and STATA software. First of all, the percentage distributions, solution percentages, and measures of central tendency and variation for each item were checked in both field test versions. This clarified the difficulty level and discrimination indices of the items, and these in turn influenced the choice of items for the main study (including, for example, the exclusion of items that exhibited a large amount of missing data in the form of *no response*).

Secondly, the tools of classical test theory were applied. In both field test versions, the inter-item correlations were calculated. The inter-item correlation measures the extent to which scores on one item are related to scores on all other items in a scale, and also the extent to which items on a scale assess the same content [28]. In addition, the reliability, as measured by Cronbach’s alpha coefficient (α), was calculated to measure the internal consistency of the items belonging to the constructed HL dimensions (scales) [29], bearing in mind that the alpha coefficient indicates how closely related a set of test items are as a group.

Thirdly, the discrimination index and difficulty level were calculated for each item using the generalized partial credit model [30]. A similar two-parameter model has been applied in PISA research since 2015 [31]. Moreover, item characteristic curves (ICC curves) were examined. In test item selection, these curves give a graphical indication of the test items, as well as information on the relationship between the ability of the testees and the probability of a testee getting the item right [32].

## 3. Results

### 3.1. Developing the HL Measurement Instrument: Through Four Phases to the Main Study

Altogether 252 students participated in the field test in the autumn of 2012. Two versions of the field test (including in total 107 different items) with four linking items were administered. The construct validity was considered to be strong, since the items were formulated on the basis of the content and goals of the HE curriculum. The content validity was ensured by selecting a large number of items from the core content of HE. The experts participating in the study judged the appropriateness and usefulness of each item to determine how accurately the measurement instrument tapped into the core content areas of the HE curriculum, the dimensions of HL, and the levels of thinking, as drawn from Bloom’s taxonomy. It was hoped that the voices of the pupils and their teachers would emerge strongly in the field test (since they had opportunities to give feedback on the test) but, in fact, only limited feedback was offered. Nevertheless, on the basis of the feedback from the field test, some changes (involving, e.g., some rephrasing of items) were implemented.

The solution percentages (i.e., the difficulty level) were checked in both field test versions. The solution percentages for the items varied from 22.4% to 99.2%. On average, the students scored 63% of the maximum points available in the field test, indicating that the difficulty level for the main study would fit well with the targeted range of 60–65% [23].

Missing information was also checked for all the items. The non-response percentage between items varied from 0 to 41.7%. The missing information related mainly to items that proved to be difficult, or that required a comparative approach. Non-functional items (e.g., by being too easy or too difficult) were removed. In selecting items for the main study, a simultaneous review was carried out, taking into account the HE curriculum, HL dimensions, the levels of thinking drawn from Bloom’s taxonomy, psychometric features of the items, the views of the expert groups, and the use of different types of item.

An aim in devising a measuring instrument is to improve reliability by ensuring consistency in the instrument’s reliability, and the repeatability of the measurement [33]. The items were made as clear and unambiguous as possible. The reliability, for both field test versions as measured by Cronbach’s Alpha coefficient (α) was at least 0.90 (version A 0.90, version B 0.92). Following the field test, items with a low discrimination index (having an inter-item correlation of less than 0.20) were removed from the main study. We allowed a few exceptions to this guideline if the content of the item was essential for constructing the scale.

**Figure 2:**
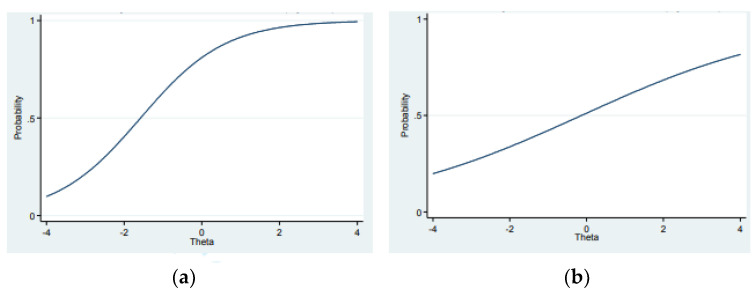
(**a**,**b**) show two different types of ICC curve. The x-axis (Theta) refers to the latent trait (ability) of the students, and the y-axis to the probability of getting a right answer.

Figure 2 shows a discriminating (left plot) and non-discriminating (right plot) item. The relationship between success in the items (correct answers) and the difficulty of the items has been modeled in the logistic function. On the left (Figure 2a, an easy item), the probability of low-ability pupils achieving a correct solution (Theta) is 0.5. A differentiating item exhibits an s-curve (as in Figure 2a). The item in the right plot (Figure 2b) is not that discriminating since, irrespective of the skill level, the probability for achieving the correct solution is very low.

For the main study, the selected items were translated into Swedish for Swedish-speaking schools. Thereafter, back-translation was performed, and the correspondence between the Swedish and Finnish texts was checked.

### 3.2. The Main Study

During the spring of 2013, EDUFI organized a national-level assessment of students’ knowledge and skills in HE at the end of the final semester in basic education (9th grade). The data were collected by obtaining a two-stage stratified random sample from 115 schools (90 Finnish-speaking and 15 Swedish-speaking schools). In total, the schools represented all the provinces, different municipality types, and schools of different types and size. Systematic sampling was used to select 9th grade pupils from each of the participating schools. The final nationally representative sample consisted of 3652 pupils.

The EDUFI assessments of learning outcomes are statutory, so the response rate is generally high. In this study, it was 96.1%. Out of the total, 52% were boys and 48% girls. About 86% studied in a Finnish-language school, and 83% spoke Finnish as their mother tongue.

The main study included 55 items (37 multiple-choice questions, 18 open-ended). The items covered a comprehensive list of health topics from the national curriculum. Ninety minutes were reserved for the pencil–paper test. Conduct of the test followed the established practices of EDUFI [23].

Table 1 shows the distribution of items, and the descriptive statistics for the main study. Numerically, the items were distributed fairly evenly over the four HE core content areas. Most of the items represented theoretical knowledge. However, there were relatively few items on critical thinking, self-awareness, and citizenship, due to the fact that they were not emphasized to the same extent as either theoretical knowledge or practical knowledge in the 2004 curriculum. Here, these HL components are treated as a single entity in Table 1 to represent HL components covering higher order thinking skills. In terms of the levels of thinking outlined in Bloom’s taxonomy, the items focused on the lowest levels, such as remembering.

In the *Health in choices in daily living* and *Understanding* dimensions, reliability remained low (α = 0.40). A more detailed analysis showed that these dimensions had more items with a low inter-item correlation (under 0.20). All these items were either right–wrong or multiple-choice items.

The construct validity and internal validity of the main study was verified via the testing in the field test. In examining the criterion validity, the pupil’s success in the assessment was compared to the grade achieved in her/his HE (as a subject). Pupils with excellent HE numerical grades (10 and 9) obtained the best results in the HL assessment (Table 2). The Pearson correlation between the HE grade and the result of the HL assessment (solution percentage) was moderately strong (*r* = 0.58, *r*-square = 34%).

Note that, according to EDUFI guidelines, the items cannot be published because of possible follow-up assessments. Examples of different kinds of items are provided in Appendix A. Both versatility and variability were taken into account when the assignments were selected for the main study.

## 4. Discussion

The overall aim was to develop a curriculum- and performance-based HL measurement instrument. The final instrument proved to be valid and reliable for measuring school-aged children’s HL in the school context. We took note of construct validity (stringent dependence of content on the curriculum to ensure construct validity), content validity (the coverage of the content to be as wide as possible), and face validity (an appropriate difficulty level), while also being aware that Messick’s [34] validity theory does not distinguish between different types of validity.

We took the view that, crucially, the instrument should reflect the measured construct (the HE national core curriculum), and hence, that construct validity can be regarded as key to evaluating the overall validity of this HL instrument.

The development steps of the HL instrument described in this paper corresponded well with the specific guidelines for the development of HL measurement as set out by Pleasant and colleagues [35]. Hence, the instrument is (i) theory-based, (ii) multidimensional in content, (iii) focused on the target group, and (iv) it prioritizes social research and public health applications, as opposed to clinical screening. The development of this measurement instrument responded to the need for a curriculum-based, objective measurement instrument for school-aged children. It also constitutes an important complementary addition to HL studies among adolescents. These have mostly emphasized the measurement of subjective HL [16] and especially functional HL [36,37], or else a single area of HL such as mental HL [38] or media HL [39]. In this sense, the study breaks new ground, both within Finland and beyond.

Many HL measurement instruments have originally been developed for adults and later adapted to meet the needs of adolescents, as in the case of REALM-TeenS [40]. HL tools for adolescents have to be less demanding, and to fit well with an adolescent’s real life. As HE (as a school subject) has primarily been developed to meet the needs of a particular age group, the objective assessment described here took the age of the participants as a starting point for the whole development process. The content areas for the instrument were based on the HE curriculum and were suitable for the target group with regard to both age and living environment. A study by Domaska and colleagues [41] has highlighted the drawbacks in using adult-oriented HL measurement instruments among adolescents. Young people were found to be unfamiliar with some terms, and they lacked experience of some health-related tasks in healthcare and disease prevention. It is essential to adjust the items in the assessment to adolescents’ state of development, and to their own experiences. This is much more likely to happen when the assessment is curriculum-based.

There is a growing understanding that HL is an important determinant of public and individual health; thus, Pleasant et al. [35] have argued for a comprehensive approach to measuring HL in the public health context, rather than in purely clinical settings. However, if one accepts that schools are a major arena for developing adolescents’ health literacy, it follows that the measurement purposes and practices should meet the expectations of the educational contexts. Measuring curriculum-based HL offers valuable information to develop evidence-informed conditions for learning, and to develop the national curriculum further. In Finland, the outcomes of the assessment were applied in developing the current national curriculum. As an example, the test showed an insufficiency in adolescents’ critical thinking skills [42], which was taken into account by strengthening the role of critical thinking in HE as a school subject, and its goals for instruction and learning in the new national curriculum.

Both HE and HL have a substantial role in health promotion. HL is also a significant skill, comprising both the goal and the result of health promotion throughout the lifespan. Hence, HL should be measured, including among adolescents, implying further the need to develop measurement instruments for young people. Finland is one of the few countries in which HL has been explicitly used as a framework for identifying and describing HE learning goals within the national core curriculum. Indeed, the detailed Finnish HE curriculum has facilitated the preparation of the measurement instrument. This can also be seen as having improved the content validity of the assessment.

### Strengths and Limitations

One strength of the current study is its multifaceted approach, and its comprehensive coverage of HE and HL. The content validity was good, insofar as the measurement instrument covered broadly the core content areas of the curriculum. This is a strength, but it was also a major challenge, given that the core content areas of the HE curriculum are so broad. Despite this breadth, the instrument must be sufficiently compact for the pupils to be able to focus on the test and answer the questions.

The other strength is the versatility of the item types. Multiple choice items have been a typical feature of HL measurement instruments [16]. However, we also used open-ended items. Such items gain more purchase on pupils’ deeper thinking. However, they are also challenging to test administrators, because of the time needed to review and score them.

The instrument described here is culture specific. It was developed to measure the learning pertaining to specific objectives, laid down in the Finnish curriculum. Hence, it would not make sense to apply the instrument to other countries as it stands, though many items would still be relevant, and other items could be substituted fairly readily.

The developed measurement instrument also has other limitations. The items were primarily guided by the HE curriculum. This resulted in the distribution of items not being entirely equivalent to the dimensions of HL, or to the levels of thinking included in Bloom’s taxonomy. The mismatch was due to a clear emphasis on competencies related to factual and practical knowledge in the 2004 HE national curriculum. Hence, the assessment had to focus on lower levels of thinking (e.g., remembering and recognizing) and the corresponding HL dimensions (theoretical and practical).

In addition, the EDUFI’s guidelines for the development process of the measurement instrument do not make room for any classical test–retest protocol. The test–retest method is one of the core methods for testing the stability and reliability of an instrument during the development process. The use of a test–retest method would have clarified some items, and further improved the reliability of the measurement instrument. It is also necessary to note that the measurement instrument itself is by no means static; indeed, it will have to be updated when the HE curriculum changes.

## 5. Conclusions

The development process of the HL measurement instrument was successful; nevertheless, it could be developed in the direction of more practical assignments, pertaining, for example, to first-aid situations, as when a pupil has to apply support to a sprained ankle. Bjørnsen and colleagues [43] have argued that knowledge does not in fact mean skills; rather, knowledge is basic to the building of skills in such a way that knowledge is applied. It is also a necessary starting point for promoting mental health among adolescents. The same point of view is relevant to the promotion of HL and HE.

As the study was the first to comprehensively investigate various forms of learning in HE, its scope and approach are unique, both nationally and internationally. Our overall conclusion is that this HL measurement instrument can provide a reliable and valid assessment of multidimensional HL in Finnish schools. The final results of this assessment offered valuable information for preparation of the subsequent HE curriculum, which was introduced in schools in 2016 [19]. Moreover, the development process described here could also be applied to other countries; hence, it has the potential to complement the field of HL assessment more broadly. 

## Figures and Tables

**Table 1:** Descriptive statistics for the main study items.

Themes	Number of Items	Max. Points	α	Solution Percentage	SD	95% Confidence Interval	
The core content areas of Health Education:							
Growth and development	14	22	0.73	58.2	18.0	57.6	58.8
Health in choices in daily living	16	22	0.40	61.5	12.3	61.1	61.9
Resources and coping skills	11	15	0.56	64.9	20.4	64.2	65.5
Health, society, and culture	14	23	0.68	53.4	18.5	52.8	54.0
The dimensions of HL:							
Theoretical knowledge	33	42	0.73	61.1	13.0	60.7	61.5
Practical knowledge	12	17	0.60	60.4	16.9	59.9	60.9
Critical thinking, self-awareness, and citizenship	10	23	0.74	54.0	21.9	53.3	54.7
The levels of thinking from Bloom’s taxonomy:							
Remembering, recognizing	31	41	0.73	63.2	13.9	62.8	63.7
Understanding	10	10	0.40	59.4	18.3	58.8	60.0
Applying	8	16	0.62	59.8	19.3	59.1	60.4
Analyzing, evaluating, creating	6	15	0.67	46.3	23.2	45.5	47.0

**Table 2:** The association between HE grades and success in the HL assessment.

Health Education numerical grade (as a school subject)	4	5	6	7	8	9	10
Solution percentage (%)	13.4	40.3	44.9	50.3	57.9	67.2	74.1

## Data Availability

As a research project for which the information in this manuscript does not include links to publicly archived analyzed materials, permission to use the material must be requested from Karvi. Data supporting the results reported in the manuscript can be obtained upon reasonable request to the corresponding author.

## Appendix A. Examples of Different Kinds of Items

**Table:**

**Example Questions (Closed-Ended)**	**Focus of the Item**	**Link to the Health Education Curriculum**
*Celiac disease is the same as a grain allergy.* *True or false?*	Health in choices in daily living Theoretical knowledge Remembering	Pupils should be able to name the most relevant diseases.
*Your godparent has high cholesterol. What should he/she do?* He/she should increase the amount of saturated fat in his/her diet. He/she should increase the amount of animal fat in his/her diet. He/she should increase the amount of unsaturated fat in his/her diet. He/she should increase the amount of hard fat in his/her diet.	Health, society, and culture Practical knowledge Application	Pupils should know the most common national diseases, and their risk factors.
*Link health troubles to their treatment. Write only one letter on each line. Note that there is one extra treatment.* Constipation ___ Neck and shoulder pain ___ Mild hypothermia ___ Heartburn ___ Mild sunstroke ___ Ear infection ___ Medication to reduce stomach acid Cooling and a cold drink Corrected ergonomics Warm clothes and a warm drink Antibiotics Fiber-rich food and an adequate fluid intake Warm drinks & anti-inflammatory medicine	Resources and coping skills Practical knowledge Understanding	Pupils should know how to make observations on their symptoms, and know the basics of the appropriate use of medicines. Pupils should be able to act appropriately in situations relevant to health and disease.
*Example questions (open-ended)*		
*How can music as a hobby promote health? Give two (2) examples, and justify your answer.*	Health, society, and culture Critical thinking Analyzing	Pupils should be able to assess the importance of culture from the perspective of health.
*What are the three dimensions of health defined by the World Health Organization? Give one example of your own health promotion choices for each health dimension.*	Resources and coping skills Self-awareness Application	Pupils should be able to weigh up the implications of lifestyle choices for their health, and give examples of day-to-day choices that promote health.
*How does society support non-smoking in Finland?*	Health, society, and culture Citizenship Analyzing	Pupils should understand the significance of rules, agreements, and trust as prerequisites for the wellbeing of communities, including society as a whole.
